# Delineating the Drivers and Functionality of Methanogenic Niches within an Arid Landfill

**DOI:** 10.1128/aem.02438-21

**Published:** 2022-04-11

**Authors:** Mark C. Reynolds, Damien Finn, Analissa F. Sarno, Richard Allen, J. David Deathrage, Rosa Krajmalnik-Brown, Hinsby Cadillo-Quiroz

**Affiliations:** a School of Life Sciences, Arizona State University, Tempe, Arizona, USA; b Biodesign Institute, Arizona State University, Tempe, Arizona, USA; c Center for Bio-mediated & Bio-inspired Geotechnics, Arizona State University, Tempe, Arizona, USA; d Salt River Landfill, Scottsdale, Arizona, USA; e Copper State Engineering Inc., Scottsdale, Arizona, USA; f School of Sustainable Engineering for the Built Environment, Arizona State University, Tempe, Arizona, USA; Royal Netherlands Institute for Sea Research

**Keywords:** landfill, leachate, methane, methanogens, niche, geochemistry, microbiome, biogeochemistry

## Abstract

Microbial communities mediate the transformation of organic matter within landfills into methane (CH_4_). Yet their ecological role in CH_4_ production is rarely evaluated. To characterize the microbiome associated with this biotransformation, the overall community and methanogenic *Archaea* were surveyed in an arid landfill using leachate collected from distinctly aged landfill cells (i.e., younger, intermediate, and older). We hypothesized that distinct methanogenic niches exist within an arid landfill, driven by geochemical gradients that developed under extended and age-dependent waste biodegradation stages. Using 16S rRNA and *mcrA* gene amplicon sequencing, we identified putative methanogenic niches as follows. The order *Methanomicrobiales* was the most abundant order in leachate from younger cells, where leachate temperature and propionate concentrations were measured at 41.8°C ± 1.7°C and 57.1 ± 10.7 mg L^−1^. In intermediate-aged cells, the family *Methanocellaceae* was identified as a putative specialist family under intermediate-temperature and -total dissolved solid (TDS) conditions, wherein samples had a higher alpha diversity index and near CH_4_ concentrations. In older-aged cells, accumulating metals and TDS supported *Methanocorpusculaceae*, “*Candidatus* Bathyarchaeota,” and “*Candidatus* Verstraetearchaeota” operational taxonomic units (OTUs). Consistent with the *mcrA* data, we assayed methanogenic activity across the age gradient through stable isotopic measurements of δ^13^C of CH_4_ and δ^13^C of CO_2_. The majority (80%) of the samples’ carbon fractionation was consistent with hydrogenotrophic methanogenesis. Together, we report age-dependent geochemical gradients detected through leachate in an arid landfill seemingly influencing CH_4_ production, niche partitioning, and methanogenic activity.

**IMPORTANCE** Microbiome analysis is becoming common in select municipal and service ecosystems, including wastewater treatment and anaerobic digestion, but its potential as a microbial-status-informative tool to promote or mitigate CH_4_ production has not yet been evaluated in landfills. Methanogenesis mediated by *Archaea* is highly active in solid-waste microbiomes but is commonly neglected in studies employing next-generation sequencing techniques. Identifying methanogenic niches within a landfill offers detail into operations that positively or negatively impact the commercial production of methane known as biomethanation. We provide evidence that the geochemistry of leachate and its microbiome can be a variable accounting for ecosystem-level (coarse) variation of CH_4_ production, where we demonstrate through independent assessments of leachate and gas collection that the functional variability of an arid landfill is linked to the composition of methanogenic *Archaea*.

## INTRODUCTION

It is estimated that 7 billion to 10 billion tons of solid waste were generated globally in 2015 (1). Nearly 70% of this waste is disposed of in a landfill, with municipal solid waste (MSW) representing the predominant fraction of landfilled waste (2). Landfilling is historically the primary route of MSW disposal. Recent models estimate that this trend will continue despite efforts to move toward alternative technologies (e.g., anaerobic digestion) that promote the recovery of useful resources, such as biogas, or biomass (3–6). Biogas capture and harvesting for methane (CH_4_), collectively termed biomethanation in industry, are proven approaches for effective waste-to-energy recovery and are estimated to be viable in approximately 18% of active or closed U.S. landfills (7, 8). Yet establishing these practices in existing sites and incorporating the required infrastructure in landfill operations remain economically challenging.

The biodegradation of MSW requires diverse and interacting populations of cellulolytic, fermentative, and methanogenic microorganisms (9, 10). The activity of these microorganisms, including those performing methanogenesis, is dependent on physical (e.g., temperature) and chemical (e.g., hydrogen partial pressure) factors (11). The prokaryotic community composition and geochemistry of MSW by-products (aqueous and gaseous) define four commonly studied phases of MSW biodegradation. As proposed elsewhere (12), MSW biodegradation starts aerobically, where polymer hydrolysis is active and most energetically favorable. The subsequent phase, anaerobic acid, is attributed to high rates of fermentation of soluble carbohydrates and proteins, where acid-tolerant methanogenic *Archaea* are known to increase sharply in biomass and metabolic activity (13, 14). The final stages represent shared, terminal steps in the biodegradation of organic matter under anaerobic conditions: accelerated and decelerated CH_4_ production. These stages are marked by a return to neutral pH and depleted concentrations of the methanogenic substrates acetate (i.e., driven in part by acetoclastic methanogenesis), hydrogen (i.e., driven in part by hydrogenotrophic methanogenesis), or other organic precursors to acetate.

Early reports of the MSW microbiome were described from leachate (i.e., liquids percolating through a waste matrix) based on culture-dependent techniques such as the most-probable-number technique (12) and culture-independent quantification of universal prokaryotic biomarker genes (e.g., 16S rRNA) (15–17). Landfills collect leachate at bottom depths, acting as a composite representation of an area determined by the vertical column and horizontal space covered by the leachate drainage network. Leachate is readily accessible, its sampling is not disruptive or costly, and its percolating nature can represent a larger pooled sampling (spatially coarse) of the landfill instead of individual depth layers of landfills. Comparisons of the microbiomes accessible through leachate (planktonic or detached cells) or solid-waste (waste-attached cells) samples have been reported using a variety of molecular approaches (14, 18, 19), supporting the use of leachate to capture general trends in the microbiome despite some discrepancies in the putative abundances of some microbial groups.

Furthermore, studies characterizing prokaryotic communities in leachate using PCR have shown that MSW landfill leachate not only is distinct from other microbiomes but also differs between landfills. Regional climate; macronutrient levels, including total phosphorus; and pH were identified as major determinants of leachate community composition (16, 17, 20). A study comparing the prokaryotic compositions of landfills across the United States revealed distinct characteristics of landfills in arid regions, including Arizona (17). For example, arid landfill microbiomes contained a low abundance of hydrolytic phyla (e.g., *Bacteroidetes* and *Firmicutes*) putatively involved in the biodegradation of organic matter (21), which can have functional consequences on CH_4_ production. Besides hydrolytic activity, limited moisture availability and diminished hydraulic conductivity can have net consequences on other aspects of landfill microbial ecology, including fewer dispersal opportunities (22) and increased desiccation and mortality-driven community shifts (23, 24). Besides the previous study by Stamps et al. (17), we have identified only one other study describing the prokaryotic composition of a single leachate sample collected from an arid Arizona landfill (25). Thus, a more comprehensive investigation into the arid MSW landfill microbiome, to better understand the microbial processes and constraints influencing methanogenesis, is needed.

Methanogenic *Archaea* are active members of the MSW microbiome. In 2018, emissions of CH_4_ from U.S. landfills were estimated at 1 billion metric tons of carbon dioxide-equivalent global warming potential (26). Despite ample evidence of methanogenic activity, the assessment of methanogenesis alongside a changing microbiome has been frequently neglected in studies of commercial landfills. Hydrogenotrophic methanogenesis, using hydrogen as an electron donor to reduce carbon dioxide, is commonly described as the predominant methanogenic metabolism from MSW material (14, 15, 27), where it also acts as an important hydrogen sink (28). Furthermore, even with increased numbers of studies reporting the complexity and succession of MSW communities (14, 29–31), few have sought to identify explicit conditions of methanogen community structure in relation to variable CH_4_ production. Shifts and maintenance of methanogenic communities, including cosmopolitan and/or rare taxa, across diverse environmental conditions are a knowledge gap not only for sustainable landfill practices but also for fundamental ecology and emerging climate feedbacks (32, 33).

Our understanding of MSW biodegradation linked to methanogenesis is improving but has been derived mainly from small-scale, laboratory-based experiments, which rarely match field conditions and natural fluctuations experienced in a landfill. Identifying predominant bioprocesses *in situ* and the environmental conditions affecting them remains a top priority to investigate, while laboratory simulations will always be needed to determine the effects of the microbiome suggested by correlations identified in field studies. The successful operation of a commercial landfill can benefit from temporal and spatial assessments of methanogenesis, yet the framework to effectively manage methanogen communities *in situ* toward CH_4_ capture or mitigation of emissions remains absent. Moreover, the advantages of microbial monitoring in landfill operation hold the potential to better inform landfill design and expansion.

In this work, we studied the leachate microbiome of an arid, commercial landfill to reveal the variable structuring of methanogenic communities and their potential implications for landfill-wide CH_4_ production and activity. We hypothesized that the prokaryotic composition of leachate and its associated geochemistry select for distinct microbial compositions and methanogenic niches contributing to landfill-wide CH_4_ production and the activity of the hydrogenotrophic methanogenesis pathway. Furthermore, the distribution of taxa across the niches can inform on which methanogenic taxa act as specialists with increased abundances under the categories’ variable geochemical gradients.

We use “niche” under the umbrella term introduced by MacArthur and Levins and used by other groups to describe individual species’ impact on resource utilization or select species’ “roles” (34, 35, 36). Specifically, we use the term to identify indicator taxa associated with certain stages of MSW biodegradation, where the abundance of operational taxonomic units (OTUs) is an indication of a taxon’s competitiveness or adaptation to local environmental conditions (37). We propose that characterizing the methanogenic microbiome throughout geochemically diverse sections of an active and expanding MSW landfill will contribute to better predict the predominant types and rates of methanogenesis at a landfill.

## RESULTS

### Discrete variations in CH_4_ production and leachate geochemistry found in cell categories throughout the landfill.

**FIG 1:**
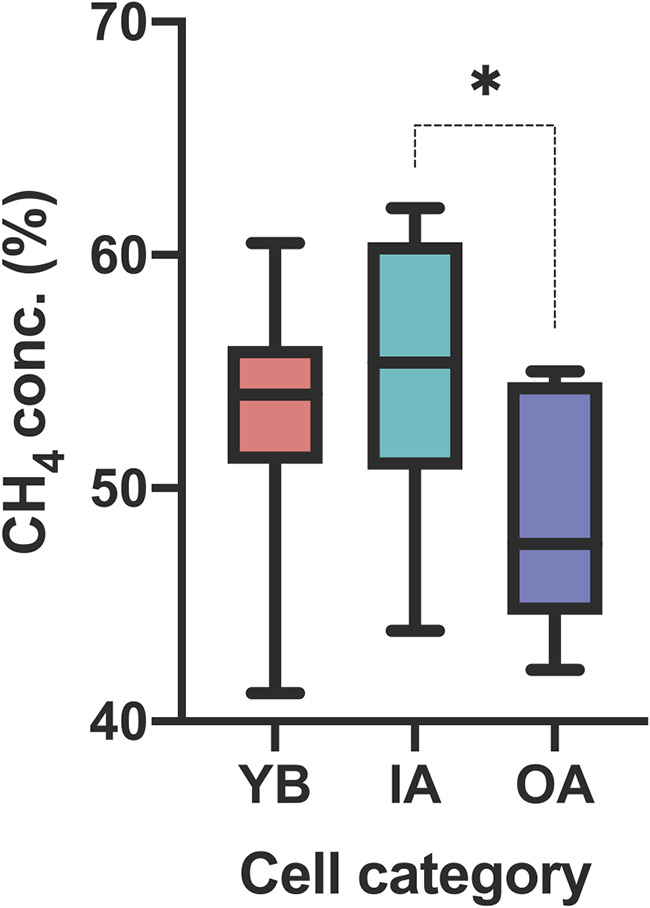
CH_4_ concentrations (*n* = 56; Kruskal-Wallis) across distinct landfill cell categories in 2018. Cell categories are young-aged, below-terrain MSW (YB); intermediate-aged, above- and below-terrain MSW (IA); and old-aged, above- and below-terrain MSW (OA). *, calculated *P* value of ≤0.05.

The overall mean CH_4_ concentration (percent, wt/wt) of landfill gas across 56 gas wells monitored throughout this study from six samplings between 2016 and 2018 at Salt River Landfill (SRL) showed a narrow range, at 54.2% ± 0.2% (*n* = 336). However, the analysis of all time points combined showed statistically significant differences for CH_4_ concentrations between the cell categories (see Fig. S1 in the supplemental material) assigned in this study (see “Study site description and categorization of landfill cells,” below). For instance, in 2018, the mean gas concentrations from the young-aged, below-terrain (YB) cells were compared to the intermediate-aged, above- and below-terrain (IA) cells or the old-aged, above- and below-terrain (OA) cells. IA cells showed the highest mean CH_4_ concentration (55.5% ± 1.1%), while OA cells had the lowest (48.5% ± 1.8%) (Fig. 1).

**FIG 2:**
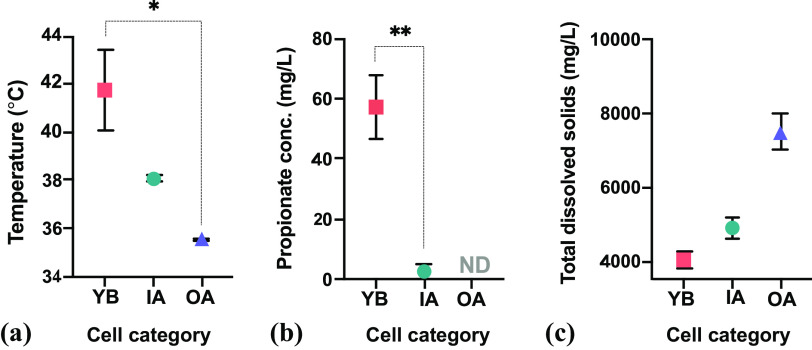
Leachate temperature (*n* = 8; Kruskal-Wallis) (a), propionate concentrations (*n* = 8; unpaired two-tailed *t* test) (b), and total dissolved solids (*n* = 7; Kruskal-Wallis) (c) across distinct landfill cell categories in 2018. For TDS analysis only, samples from leachate pump 10 (LP10) and LP11 (IA leachate) were studied as composite samples due to low leachate yields. Cell categories are young-aged, below-terrain MSW (YB); intermediate-aged, above- and below-terrain MSW (IA); and old-aged, above- and below-terrain MSW (OA). ND, below the detection level. *, calculated *P* value of ≤0.05; **, calculated *P* value of ≤0.002.

To delineate geochemical variations across cell categories, we screened leachate samples by standard methods and other detection tests (Table S1) spanning multiple organic (38) and inorganic (18) analyte measurements frequently required in landfill regulatory monitoring. Of these, three parameters (temperature, propionate concentrations, and total dissolved solids [TDS]) displayed statistically significant variation in both the 2018 samplings (Fig. 2) and a nonparametric mixed-effects model between October 2016 and 2018 (not shown) between all three cell categories. Acetate was undetected (<2.5-mg/L detection limit) in all 2018 samples and was detected only in October 2016 at 5.1 ± 2.1 mg/L within two categories (YB and IA) (*n* = 6).

In leachate samples from 2018, barium concentrations (Fig. S2), trace metals, and volatile organics measured above the analytical detection limit were variable but not statistically significant under *post hoc* pairwise comparisons. Barium concentrations (1.7 ± 0.09 mg L^−1^) and total detectable trace metals were highest in OA leachate (3.2 ± 0.08 mg L^−1^). Arsenic and nickel were present in all cell categories at <30 μg L^−1^. Volatile organics measured above the analytical detection limit were highest in IA leachate (259.7 ± 49.80 mg L^−1^) and predominated by mixed xylenes (*m*-, *o*-, and *p*-xylenes).

### Overall prokaryotic communities were structured by cell categories derived from age and influence of above-terrain MSW disposal.

**FIG 3:**
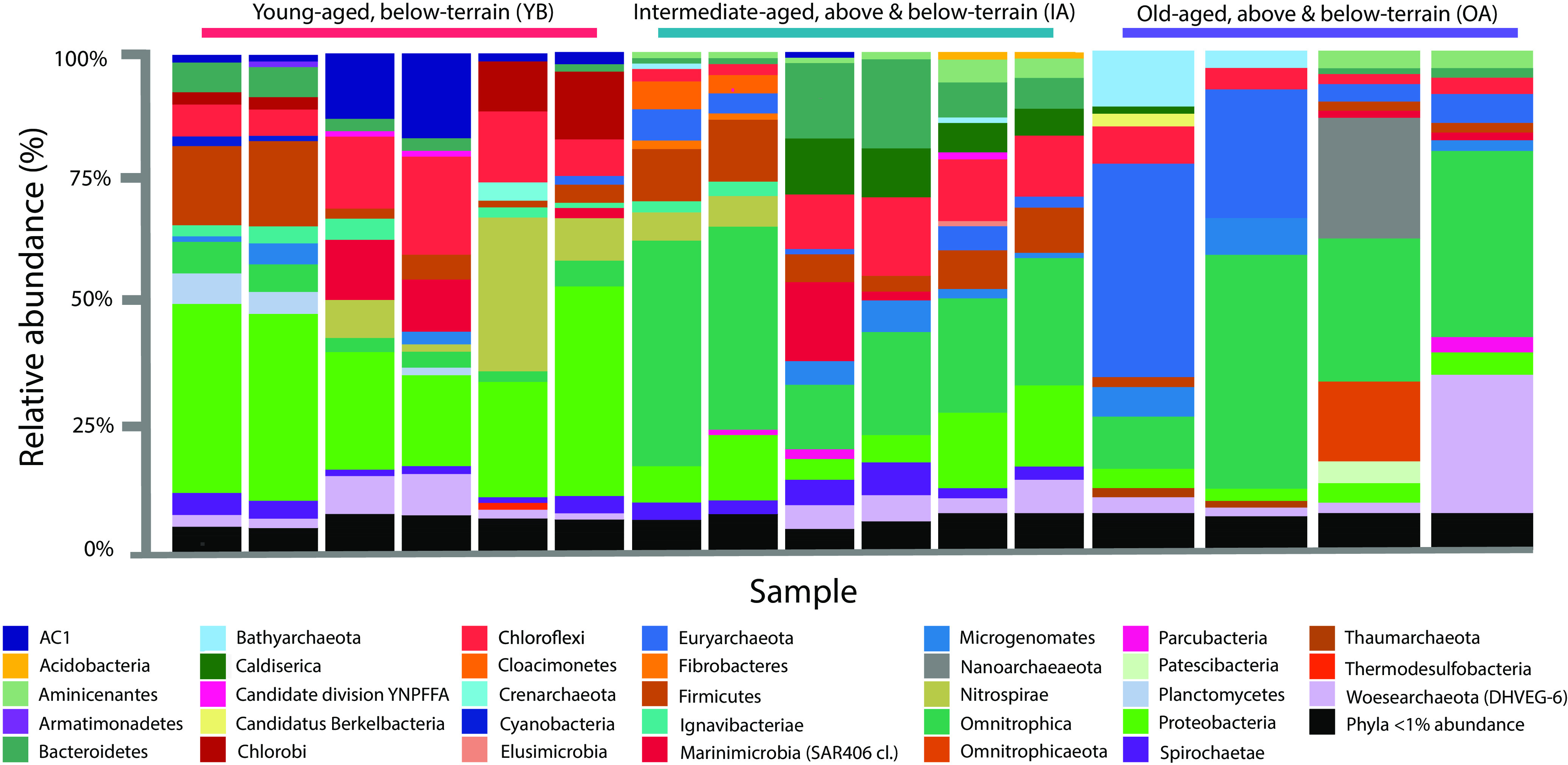
Leachate phylum-level total prokaryotic composition of distinct cell categories in 2018 derived from next-generation sequencing of the 16S rRNA gene and naive Bayes taxonomic classification. Two replicates per sample are shown.

The 16S rRNA marker sequencing of the overall bacterial and archaeal communities from eight fluid-yielding leachate pumps in June 2018 resulted in 421,552 reads, sorted as 2,785 features (absolute sequence variants [ASVs]). The majority of the taxonomic assignments had high-confidence scores; however, two neighboring OA leachate pumps showed a significant fraction of unassignable reads (23.6% ± 3.1% and 5.7% ± 3.4%) using the SILVA 132 database (Fig. 3). OA leachate also had the highest, statistically significant, proportion of archaeal reads across cell categories (*P* < 0.05 by ANCOM [analysis of the composition of microbiomes]). One OA sample had 45.5% ± 9.8% of its reads assigned to the archaeal phyla *Euryarchaeota* and “*Candidatus* Bathyarchaeota,” while the other OA sample contained the greatest number of reads mapping to “*Candidatus* Woesearchaeota” (Deep Sea Hydrothermal Vent Group 6 [DHVEG-6]), at an 11.6% ± 10.1% relative abundance. Compared to all OA samples, archaeal reads were only 6.1% and 8.4% abundant in samples collected from YB and IA cells, respectively, nearly 5 times less prevalent. A few exceptions were observed, with one YB sample harboring 7.9% ± 0.3% “*Candidatus* Woesearchaeota.”

The patterns of bacterial composition were distinct from those of *Archaea*. Bacterial composition instead showed that communities from leachate generated from exclusively below-terrain MSW (YB cells) were distinct from those communities receiving leachate from both above- and below-terrain MSW (OA and IA cells). Phylum-level differential testing by ANCOM by cell category revealed that *Bacteria* within “*Candidatus* Cloacimonetes” and *Chlorobi* were differentially abundant in YB leachate (*P* < 0.05). Although not statistically significant by ANCOM, YB leachate also had the highest relative abundance of *Proteobacteria* (29.6% ± 3.8%), while this phylum was typically a minor fraction of the leachate from cell categories with above-terrain MSW disposal. In both IA and OA leachates, instead, the following phyla were uniquely abundant: “*Candidatus* Omnitrophica/Omnitrophicaeota” (OP3), “*Candidatus* Microgenomates” (OP11), “*Candidatus* Caldiserica” (OP5), “*Candidatus* Aminicenantes (OP8),” “*Candidatus* Berkelbacteria,” and *Fibrobacteres*.

### *Methanomicrobiales* were most abundant in young-aged, below-terrain leachate, while *mcrA* alpha diversity increased in intermediate-aged, above- and below-terrain leachate.

**FIG 4:**
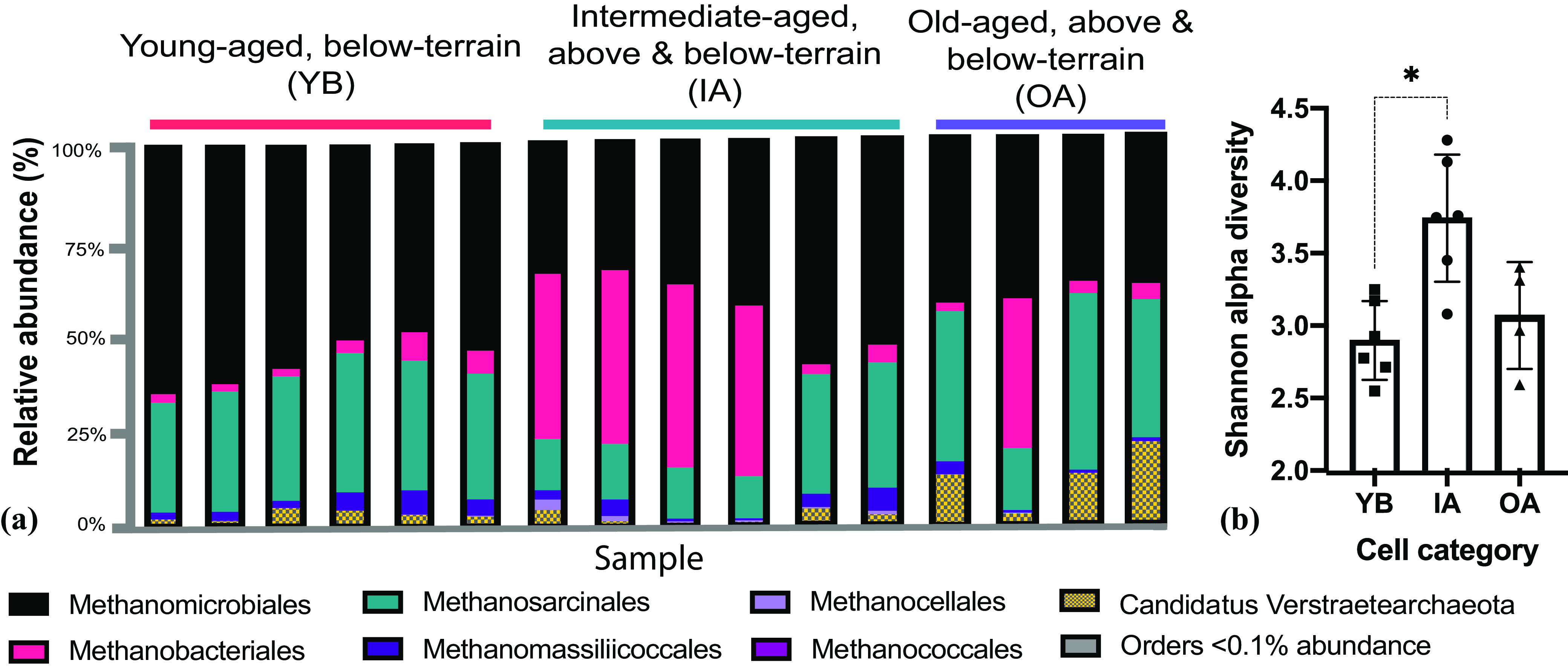
Leachate order-level methanogenic *Archaea* composition derived from next-generation sequencing of the *mcrA* gene and amino-acid-based nearest-neighbor classification (a) and Shannon alpha diversity from distinct cells categories (b) in 2018. Analysis of *mcrA* translated to amino acids resulted in 302,897 reads, comprising 4,541 features (OTUs). Two replicates per sample are shown.

We evaluated methanogen community composition by comparing the environmental sequences of the ecofunctional genetic marker *mcrA* against a custom reference database (Data Set S3) via nearest-neighbor classification with high-confidence scores (Data Set S2). Analysis of *mcrA* translated to amino acids resulted in 302,897 reads, comprising 4,541 features (OTUs). Reads were assigned to six out of the seven currently recognized euryarchaeal methanogenic orders (Fig. 4a), followed by clustering at 86% percent identity (39). Five orders were present in every leachate sample. The hydrogenotrophic order *Methanomicrobiales* was the most abundant, followed by the metabolically versatile *Methanosarcinales* and *Methanobacteriales* third. *Methanomassiliicoccales*, an obligate methylotrophic order (40), had a modest representation at a <10% relative abundance in all samples. *Methanococcales* were detected at a minimum abundance (≤9 reads per sample) across leachate samples and were considered to be only a minor group. Leachate from intermediate-aged, above- and below-terrain (IA) cells had significantly higher Shannon alpha diversity than young-aged, below-terrain (YB) samples (Fig. 4b) when samples were rarefied to ∼3,000 sequences per sample. Specifically, the order *Methanocellales* was significantly overrepresented in IA cells (*P* = 0.019 by a Kruskal-Wallis test; *P* < 0.05 by ANCOM).

The relative abundances of *Methanomicrobiales* and *Methanobacteriales* revealed shifts in the methanogenic community structure related to the cell categories and distinct from the community structure seen in *Bacteria*. The order *Methanomicrobiales* was the predominant order for all cell categories, and total reads were statistically highest within YB leachate, at 56.5% ± 2.7%. Meanwhile, total *Methanobacteriales* reads increased in abundance by more than 5 times within IA leachate in comparison to its chronologically preceding category, YB. A shifting composition from *Methanomicrobiales* to *Methanobacteriales* is also evident in one of the OA samples.

OTUs assigned to non-*Euryarchaea*, “*Candidatus* Verstraetearchaeota,” were highly abundant members of the methanogenic community, with a mean of 4.8% ± 1.4% among all leachate samples and their replicates. The relative abundance of “*Candidatus* Verstraetearchaeota” was highest in OA leachate (12.3% ± 3.7%). All “*Candidatus* Verstraetearchaeota” reads were classified with suitable confidence (Data Set S2) at 85.8% ± 0.04% and were represented as a monophyletic clade with high support in complementary Bayesian phylogenetic analysis (Fig. S4). This clade was most closely related to *Methanomassiliicoccales* and included a reference metagenome-assembled genome collected from a hot spring (41). No *mcrA* reads were classified as putative anaerobic methanotrophic *Archaea* (ANME) from the ANME-1 or ANME-2 clades, although sequences affiliated with these elusive groups have been recovered in other landfill characterization work as 16S rRNA sequences (42) using distinct primers compared to the work conducted here.

### Combined analysis of geochemistry and microbial data supported contrasting methanogenic niches within leachate from cell categories.

**FIG 5:**
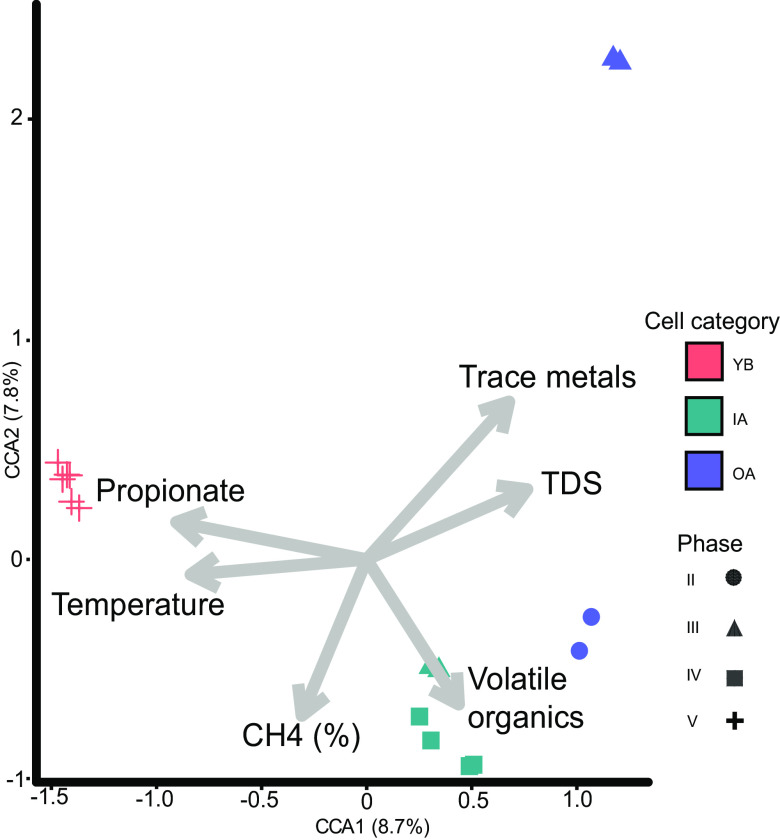
Canonical correspondence analysis (CCA) ordination of family-level *mcrA* gene abundance data from 2018 to visualize the landfill’s methanogenic niches using Hellinger-transformed Bray-Curtis distances. PERMANOVA showed statistically significant differences in the structure of the methanogenic communities across the assigned landfill cell categories (*P* < 0.001).

Permutational multivariate analysis of variance (PERMANOVA) showed statistically significant differences in the structures of the methanogenic communities across the assigned landfill cell categories (*P* < 0.001 by PERMANOVA). Furthermore, we completed a canonical correspondence analysis (CCA) ordination using Hellinger-transformed frequencies under Bray-Curtis distances. The statistically significant variables leachate geochemistry for the 2018 sampling (i.e., temperature, TDS, propionate, trace metals, and gas chromatography-mass spectrometry [GC-MS]-measured volatile organics) and CH_4_ gas concentrations nearby to the leachate sampled from the drainage network were incorporated as additional ordination loadings (Fig. 5). Differences in the methanogen community compositions of OA leachate were associated with elevated TDS and trace metals, including barium, arsenic, and nickel. IA leachate was positively correlated with the highest CH_4_ concentrations and particularly volatile organic concentrations composed predominately of mixed xylenes and ethylbenzene. In YB leachate, loadings of temperature and propionate were the strongest contributors to the samples’ dissimilarity. The CCA performed on the 16S rRNA (Fig. S5) gene depicts similar clustering patterns as described above. In the methanogen communities, the propionate concentration was the strongest contributor to component axis CCA1, and trace metals were the strongest contributor to CCA2. However, in 16S rRNA communities, leachate TDS was critical for the separation of ordination points in CCA1, while the volatile organic loading had the strongest contribution to CCA2.

To identify if highly abundant members of the ordination clusters overlapped in preferences to fill putative niches, we calculated Feinsinger’s proportional similarity (FPS) of resource utilization (43) in our family-level *mcrA* data set using the R package MicroNiche (44). MicroNiche calculates ecological niche breadth from an abundance table along with outputting the limit of quantification (LOQ), null distributions, and the 5% and 95% quartiles of the distributions. To conservatively flag putative specialists, family-collapsed taxa were designated if their calculated FPS value was above the 95% or below the 5% quartile threshold. Under the FPS ecological niche breadth calculation, taxa may act as specialists given a propensity to be highly abundant in relation to a high (i.e., the taxon has a high FPS value) or low (i.e., the taxon has a low FPS value) value of an analyte measurement.

**FIG 6:**
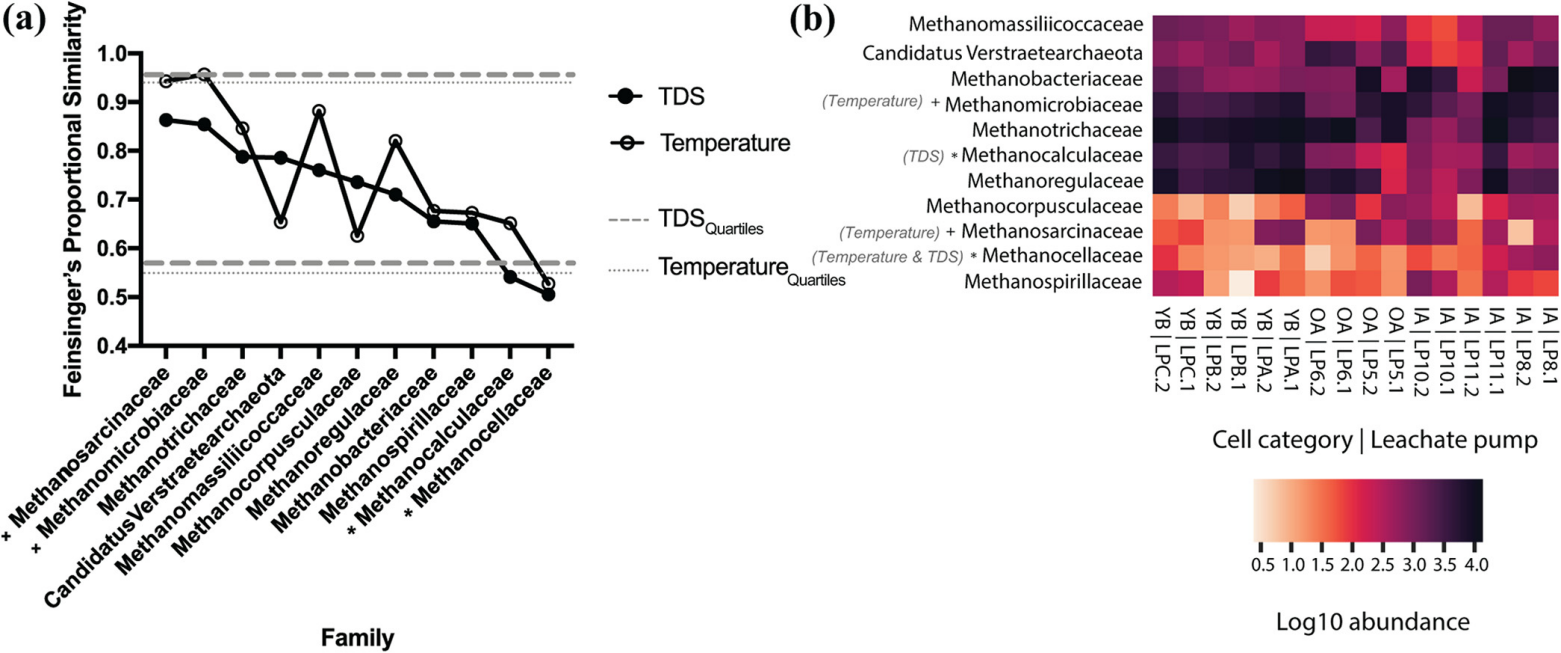
Niche breadth estimations using Feinsinger’s proportional similarity (FPS) considering leachate TDS and temperature. The designation of putative specialist families under low- or high-analyte measurements was determined against the TDS (closed circles) or temperature (open circles) 5% or 95% quartile (lines) (a), respectively. Abundance heatmap for methanogenic families analyzed in panel a under log_10_ transformation (b). Only the family *Methanocellaceae* was identified as a specialist family of both intermediate-TDS and -temperature conditions from FPS analyses. Leachate samples according to their collection source (“pump”) are labeled. +, positively correlated family; *, negatively correlated family.

MicroNiche testing was conducted individually with selected analytes like TDS and temperature, which had shown clear variation gradients and were identified as drivers in the CCA. Since propionate concentrations had several values below the detection level, we excluded propionate from the MicroNiche analysis. Figure 6a presents the niche breadths (as FPS values) of the 11 families above the LOQ in descending order of TDS proportional similarity (FPS) values.

Two specialist families (*Methanocalculaceae* and *Methanocellaceae*) were identified that showed significantly lower niche breadth FPS scores under optimal TDS and/or temperature than the mean niche breadth scores of the null-hypothesis model. This is supported by the increased abundance of *Methanocalculaceae* in the low-concentrated TDS leachate (YB) and by the negative correlation of this family’s read abundance (*r*^2^ = 0.3505; *P* < 0.05) with TDS levels (Fig. S8a). The temperature-based analysis also showed *Methanocellaceae* as specialists with significantly lower niche breadth scores following a negative binomial distribution where reads increased at moderately mesophilic temperatures (mean temperature of 38.1°C) (*n* = 6) but not at higher or lower temperatures. Meanwhile, *Methanosarcinaceae* and *Methanomicrobiaceae* were also specialists that increased at intermediate temperatures, but the niche breadths of these families were not significantly different from the null hypothesis.

To further evaluate the MicroNiche results, we visualized the log_10_-transformed frequencies of the 11 families with LOQ support. Here, the intermediate-temperature, specialist families *Methanosarcinaceae* and *Methanomicrobiaceae* are found at the highest abundance in the IA categories, while the specialist families *Methanocellaceae* (i.e., intermediate-TDS and -temperature specialist) (*P* < 0.05) and *Methanocalculaceae* (i.e., low-TDS specialist) (*P* < 0.001) share modest enrichment in abundance within the IA and YB categories, respectively (Fig. 6b).

### Functional evidence that methanogenesis varied across landfill cells.

We measured gaseous carbon stable-isotope values from the deepest gas wells of the landfill to assess whether CH_4_ measurements reflected the niche patterns identified from the *mcrA* CCA. For technical reasons, in 2018, gas samples were collected within the proximity of only two of the three cell categories (YB and IA) (*n* = 6), but an additional effort in May 2020 sampled all three categories (*n* = 8).

**FIG 7:**
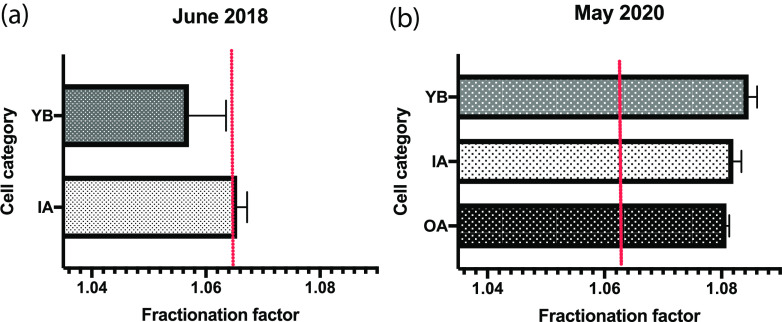
Stable-isotope ^13^C fractionation derived from δ^13^C of CH_4_ and δ^13^C of CO_2_ across cell categories. Shown are data for 2018 samplings, including IA and YB (OA was not sampled) (a), and May 2020 sampling, including all landfill cell categories (b). Cell categories are young-aged, below-terrain MSW (YB); intermediate-aged, above- and below-terrain MSW (IA); and old-aged, above- and below-terrain MSW (OA).

Previous reviews have highlighted the utility of stable carbon fractionation factors to delineate highly active methanogenic pathways (45, 46). A fractionation factor (α_mean_) of ≥1.065 has been shown to be representative of hydrogenotrophic methanogenesis, and one of ≤1.055 has been shown to be representative of heterotrophic methanogenesis (e.g., acetoclastic or methylotrophic). The fractionation factors that we report provide evidence of hydrogenotrophic methanogenesis acting as the dominant pathway in 2018 and verified during an extra 2020 sampling of the landfill. For 2018 samples (Fig. 7a), gas from the IA cells (α_mean_ = 1.065 ± 0.002; *n* = 3) was more enriched than that from the YB cells (α_mean_ = 1.057 ± 0.007; *n* = 3), pointing to the potential contribution of acetoclastic and/or methylotrophic methanogenesis in the YB cell. Meanwhile, in May 2020 samples (Fig. 7b), all cell categories’ fractionation patterns aligned with hydrogenotrophic methanogenesis, with YB’s fractionation now being the highest. Differences among cell categories were negligible. YB cells at this time point showed the highest fractionation (α_mean_ = 1.084 ± 0.002; *n* = 3), and OA leachate had the lowest (α_mean_ = 1.081 ± 0.001; *n* = 2). Gas associated with IA leachate had middle-range fractionation activity (α_mean_ = 1.082 ± 0.002; *n* = 3).

## DISCUSSION

### Delineation and characteristics of niches in a landfill.

This study investigated how ecosystem-level prokaryotic composition, diversity, and geochemistry of leachate can influence methanogenic niches and the functional potential of CH_4_ production in an arid MSW landfill. Three landfill niches were putatively identified throughout SRL that gave rise to distinct microbial community assemblages (Fig. 5; see also Fig. S3a and b and Fig. S5 in the supplemental material). The microbial community structure in leachate corresponded well to the categorization of the landfill cells based on the estimated time since the initial MSW disposal and the influence of above-terrain MSW disposal. Our multifaceted analysis of gas and leachate from YB, IA, and OA cells provided a framework to explore the functional significance of their methanogenic niches. For instance, Fig. 5 demonstrates that the dissimilarity of the YB methanogenic community is most influenced by leachate propionate and temperature, the IA methanogenic community is most influenced by the CH_4_ concentration and leachate volatile organics, and the OA methanogenic community is most influenced by leachate TDS and metals. Our ordination delineating methanogenic niches presented in Fig. 5 contains only loadings that were significant in either a 2018 nonparametric analysis of variance or an October 2016–2018 nonparametric mixed-effects model. We note that this is not a complete set of niche-shaping environmental conditions present in SRL leachate. For example, we do not report on the dissolved concentrations of gases of interest or major ionic species (nitrogenous and phosphorus compounds), the latter due to unavailable instrumentation. Oxygen was measured in low concentrations in the landfill gas reported in Fig. 1 (0 to 4% range) and was not significant by cell category (not shown). Similarly, although pH has been known to influence the community composition of methanogenic *Archaea* in a multilandfill study conducted in China (20), our study reveals it to be a less important niche shaper in the arid leachate of SRL where values were narrow and stable (the pH ranged from 6.44 to 6.76 among all samples) (Table S1).

The variable structuring of methanogenic communities in SRL leachate points to their potential contributions to coarse-scale CH_4_ production, albeit such a process is complex and affected by structural and gas collection management (47) as well as microbial activity in a landfill (10). Figure 1 show that during 2018 sampling, the highest median CH_4_ concentrations were in IA cells, and the lowest were in OA cells. However, site-wide concentrations ranged from 68.6% to 37.7%, with a mean of 54.2% ± 0.2%, between our six samplings, which is commonly observed in landfills (10, 12). This broad variation can be attributed to a large number of operational practices in the landfill (reviewed in reference 47), including manual adjustment of the vacuum in gas extraction wells, pointing to the need to evaluate the effects of operational practices on methanogenic communities in landfill CH_4_ production. Another source of variation for gas production, particularly in an arid landfill, is moisture distribution. An uneven distribution of liquids can lead to “cold spots” with dry and limited waste decomposition or “hot spots” with wet anaerobic decomposing regions. Leachate sampling cannot account for dry spots; thus, solid-waste sampling will be needed to evaluate dry sections. An uneven moisture distribution can lead to diminished nutrient transport and microbial dispersal. Although our data point to combined age and above-terrain influences, SRL’s meaningful intrasite community composition may additionally be an outcome of moisture-limited conditions. In the 60 days leading up to each of our samplings, we evidenced scarce rainfall rolling averages (12.5 ± 5.33 mm), and many pumps (>50%) at SRL did not yield leachate in the 2018 sampling.

Importantly, while the potential contribution of the landfill cells to the CH_4_ concentration varied across our six samplings from 2016 to 2018 (Fig. S1), total prokaryotic (Fig. S3a) and methanogenic (Fig. S3b) communities in YB and IA leachates were seemingly stable in their composition and their differences among landfill cells (*P* < 0.001 by Adonis PERMANOVA of 16S rRNA gene and *mcrA* gene data sets). This stable coarse composition provides evidence of the potential impact of long-term arid conditions on landfill leachate biology.

### Patterns and contributions of overall microbial communities and methanogens.

The combined assessment of our data offers support that IA and OA leachates harbor conditions more similar to one another than to YB. This relationship can be seen in the leachate’s geochemistry (Fig. 2), microbiome (Fig. 3 and Fig. 6b), and gaseous stable isotopic carbon fractionation (Fig. 7a and b) data sets. YB and OA cells have a ∼16-year difference since their initial below-terrain MSW disposal date, and YB and IA have an 11-year difference.

YB cells may offer conditions typically affiliated with active hydrolysis, improved hydraulic conductivity (48), and fermentation coupled to methanogenesis (29) relative to the IA and OA cells at SRL. For instance, given the high abundances of *Proteobacteria* and *Bacteria* with known nitrifying traits (*Nitrospirae*) (Fig. 3) and the statistically different abundances of obligate, acetoclastic *Methanothrix* species OTUs (7) and the metabolically diverse *Rhodocyclaceae* (Table S2), along with elevated propionate concentrations (Fig. 2b), we propose that YB leachate holds a niche that has not yet reached a stable or decelerated methanogenic phase in canonical MSW biodegradation succession (29, 30). Instead, its microbiome is likely transitioning or recently transitioned from the anaerobic acid stage to the accelerated CH_4_ production stage. Although not captured in our data set, the magnitude of organic acid accumulation and particularly the acetic acid end products from primary (sugar-driven) and secondary acetogenic (syntrophy-driven) activities can partially explain why anaerobic acid-stage microbiomes are reported to have the lowest prokaryotic richness. Mass balance calculations using volatile solid suspension concentrations have shown higher specific growth rates (0.03 to 0.05 day^−1^) in obligate anaerobic groups, including OTUs assigned to *Methanothrix* (formerly *Methanosaeta*), that are unequivocally involved in the anaerobic digestion process (49) but challenge findings of *in vitro* studies.

In contrast to YB, the higher TDS levels and total metal concentrations observed in OA leachate and the elevated volatile organics in IA leachate suggest deviations from biological optima, possibly due to diminishing resources and/or toxicity from chemically leached by-products (50). IA and OA cells have older MSW in place below terrain. The prokaryotic composition of this leachate, which was influenced not only by time but also by the above-terrain disposal of MSW, revealed potentially specialized prokaryotic taxa. In IA and OA leachates, phyla that are typically associated with biodegradation (e.g., *Bacteroidetes*, *Firmicutes*, and *Proteobacteria*, etc.) were underrepresented (<30% abundance). Instead, high abundances of *Archaea* and previously reported chemoheterotrophic *Bacteria* were detected, including members of “*Candidatus* Omnitrophica/Omnitrophicaeota” (also known as OP3), “*Candidatus* Aminicenantes” (also known as OP8), and “*Candidatus* Caldiserica” (also known as OP5) (Fig. 3).

SRL leachate samples were comparable in prokaryotic richness to values observed in the multilandfill data set of Stamps et al. (17) and other recent studies of MSW microbiomes (30, 51). In comparison to the two Arizona landfills in the study by Stamps et al., reads classified as belonging to the “*Candidatus* Omnitrophica” (also known as OP3) candidate phylum were 5- to 10-fold less frequent than in our data set. Meanwhile, reads assigned to the phylum *Chlorobi* were similarly abundant (0 to 10%) between studies. As more studies become available, the core or malleable microbiomes of arid and not-arid landfills can be identified (52), and the variations listed above can be better explained. For instance, compared to two studies on microbiome diversity in landfills in China, our results highlight that arid landfill conditions select distinct methanogenic communities, including genera such as *Methanoculleus*, *Methanocorpusculum*, and *Thermoplasmata*, compared to those in temperate or subtropical climates (16, 53).

Several adaptations can be expected by the leachate and/or solid-attached microbiome in arid climates. The arid conditions at SRL could select for a large fraction of compatible solute-forming (54), desiccant-resistant (55), spore-forming (56), or dormant (57, 58) microbes that can require rehydration for revival to an active cellular state. Cellulose, as a key component of MSW, is very hydrophilic but can be dried with ease under a vacuum. Such “wetting”/“drying” events may be common in arid landfills and could generate dissimilarity of their prokaryotic microbiome compositions as reported in comparison to other landfills in humid or temperate climates (16, 17, 20).

Methanogens are in most cases dependent on bacterial metabolism and interactions for their functions (59). Thus, their functional properties are likely to be influenced by the enrichment of chemoheterotrophic microorganisms in IA and OA leachates. While *mcrA* OTUs classified as *Methanomicrobiales* were the most abundant throughout the landfill’s leachate and particularly in YB leachate, OTUs of *Methanobacteriaceae* were identified to play an important role in the variance observed between the cell categories’ methanogenic communities due to their high abundance within IA cells and one additional OA sample. An increased abundance of *Methanobacteriaceae* has been reported in several other studies of anaerobic ecosystems, including a humid landfill (16), a continuously fed experimental bioreactor fed with acetate followed by acetate and hydrogen (60), and minerotrophic tropical peatlands (61).

Using our *mcrA* data set, we calculated ecological niche breadth scores to assess which leachate methanogenic families (*n* = 23) were specialists under the geochemical pressures of temperature and TDS (Fig. 6a). In our two single variate results, *Methanomicrobiaceae* and *Methanosarcinaceae* were identified as intermediate-temperature specialists that were most competitive at around 38°C. *Methanocalculaceae* were identified as low-TDS (i.e., <5,000 mg/L) specialists, and *Methanocellaceae* were identified as intermediate-temperature (i.e., <40°C) and -TDS (i.e., <5,000 mg/L) specialists, both with leachate niche breadths that were significantly lower than those offered by the null-hypothesis model. To support the niche index results, Fig. 6b reports the log_10_ frequencies of specialist families and others analyzed using MicroNiche. For example, high TDS in leachate might be unfavorable to members of the *Methanocalculaceae* given that their highest abundance was observed in YB cells. The TDS optimum described contrasts with the typical niche specialization seen in type strains of this family isolated from a soda lake (62) and an oil well (63). Yet Methanocalculus pumilus strain MHT-1^T^ was isolated from leachate collected from a seaport landfill with an optimum sodium chloride concentration that was 5- to 10-fold lower than that for Methanocalculus haloterans (64). Meanwhile, *Methanocellaceae* of IA leachate seem to be able to tolerate higher TDS concentrations but contain lower abundances in OA leachate where TDS exceeds 5,000 mg/L (Fig. 2c). Interestingly, *Methanocellaceae* also appear to be outcompeted in leachate where temperatures are higher than 40°C but also where temperatures are lower than 38°C (Fig. 2a). Members of the *Methanocellaceae* have been reported to have a wide temperature tolerance (i.e., 35°C to 55°C) (65) and have been isolated from both mesophilic and thermophilic rice field soils (66, 67). We were unable to locate the putative impacts of TDS on the generation of *Methanocellaceae* niches, but another study reported increased alpha diversity and evenness of *Archaea*, including methanogens, under increased TDS concentrations (68). A caveat to consider in this analysis is that the relative abundance of DNA reads does not necessarily translate into equivalent activity. This is important because conditions for the detection of activity are a highly desirable component of ecological niche investigations, yet DNA-based studies are reported more commonly (69–71). Taken together, our results converge with those of similar studies showing that as the geochemical properties of arid landfill leachate change over time, selection pressure alters the competitiveness of the predominant taxa contributing to landfill-wide CH_4_ production.

Relative to 16S rRNA gene-based profiling, sequencing of the *mcrA* gene offers better group-specific sampling and higher confidence in ecofunctional metabolic affiliations (e.g., acetoclastic or hydrogenotrophic methanogenesis) and amino-acid-based taxonomic assignments. In fact, *mcrA* was able to detect members of the recently proposed order *Methanomassiliicoccales* and phylum “*Candidatus* Verstraetearchaeota,” which have been implicated in methylotrophic and/or hydrogenotrophic methanogenesis (39, 72, 73). The observed significant (up to 22% relative abundance) and consistent presence (all 2018 and 2016–2017 samples) of “*Ca.* Verstraetearchaeota” in landfill leachate (Fig. 4a), to our knowledge, is the first report of this putative methylotrophic methanogen in landfills (38). Our tests regressing the read abundances of “*Ca.* Verstraetearchaeota” with leachate parameters found a significant positive correlation only with TDS concentrations (*R*^2^ = 0.32) (Fig. S8b) and a negative correlation with temperature (*R*^2^ = 0.23) (not shown). Our observation of the presence of “*Ca.* Verstraetearchaeota” in a leachate environment is supported by previous observations (and metagenome-assembled genomes) in anaerobic digesters (72). The correlation with TDS is likely related to the availability of nutrients in general as this group is predicted to carry out methanogenesis but also fermentation potentially using lipids, amino acids, and sugars (38), while an inverse association with temperature is contradictory to their observations in hot springs (41), although mesophilic and thermophilic members have been commonly observed within other methanogenic phyla (74). The role and adaptation of “*Ca.* Verstraetearchaeota” in landfills require further efforts. We also explored whether anaerobic methane-oxidizing organisms like the ANME-1 or ANME-2 clades were present in *mcrA* sequences, but no read was detected.

### Functional evidence of hydrogenotrophic methanogenesis in SRL.

The taxonomic assignment of our *mcrA* gene reads suggested that hydrogenotrophic methanogens are highly predominant at SRL. To evaluate the functional outcomes of methanogenic niche differentiation at SRL, hydrogenotrophic methanogenic activity was estimated through calculations of fractionation factors (α). Fractionation factors can delineate upper and lower limits representative of acetoclastic (α ≤ 1.055) or hydrogenotrophic (α ≥ 1.065) methanogenesis and have been verified in numerous pure-culture and environmental reports (75). Analysis of gas wells of landfill regions associated with varying age showed that hydrogenotrophic methanogenesis was the primary active methanogenic pathway (Fig. 7). Gas samples from the YB cell in 2018 were the only instance where a gas signature was observed with fractionation reflecting ranges for acetoclastic or methylotrophic methanogenesis, while in May 2020, YB demonstrated fractionation representative of hydrogenotrophic methanogenesis, which could be explained by the leachate methanogenic community shifting away from dependence on organic acids.

### Implications of microbial and methanogenic niches in landfills.

The results and framework of this study should be confirmed by experimental manipulation of excavated solid waste prior to using leachate community changes to inform or diagnose the attached solid-waste community in a landfill. Yet our results immediately demonstrate that the ecological succession of important prokaryotes or methanogenic *Archaea* can be characterized using landfill leachate across a landfill with a large geographical area. The results and framework of this study could be used to direct efforts to accurately elucidate the relationships between abundant methanogenic and/or prokaryotic taxa and CH_4_ production in solid-waste microbiomes. This study identified variations in leachate geochemistry, leachate microbiome representation, and landfill gas CH_4_ concentrations across space and time within a single landfill and characterized these influences on ecosystem-wide biomethanation. Previously proposed and recent efforts to accelerate MSW bioprocesses within a landfill, all of which can be argued to depend on the enrichment of a fastidious methanogenic community, can benefit from the microbiome characterization and correlations offered in this study. Accordingly, a developing interest to include microbiome monitoring in landfill operations can potentially promote more waste disposal and settling (by higher degradation), land use turnover, and energy recovery in active, closed, or planned landfills.

## MATERIALS AND METHODS

### Study site description and categorization of landfill cells.

**FIG 8:**
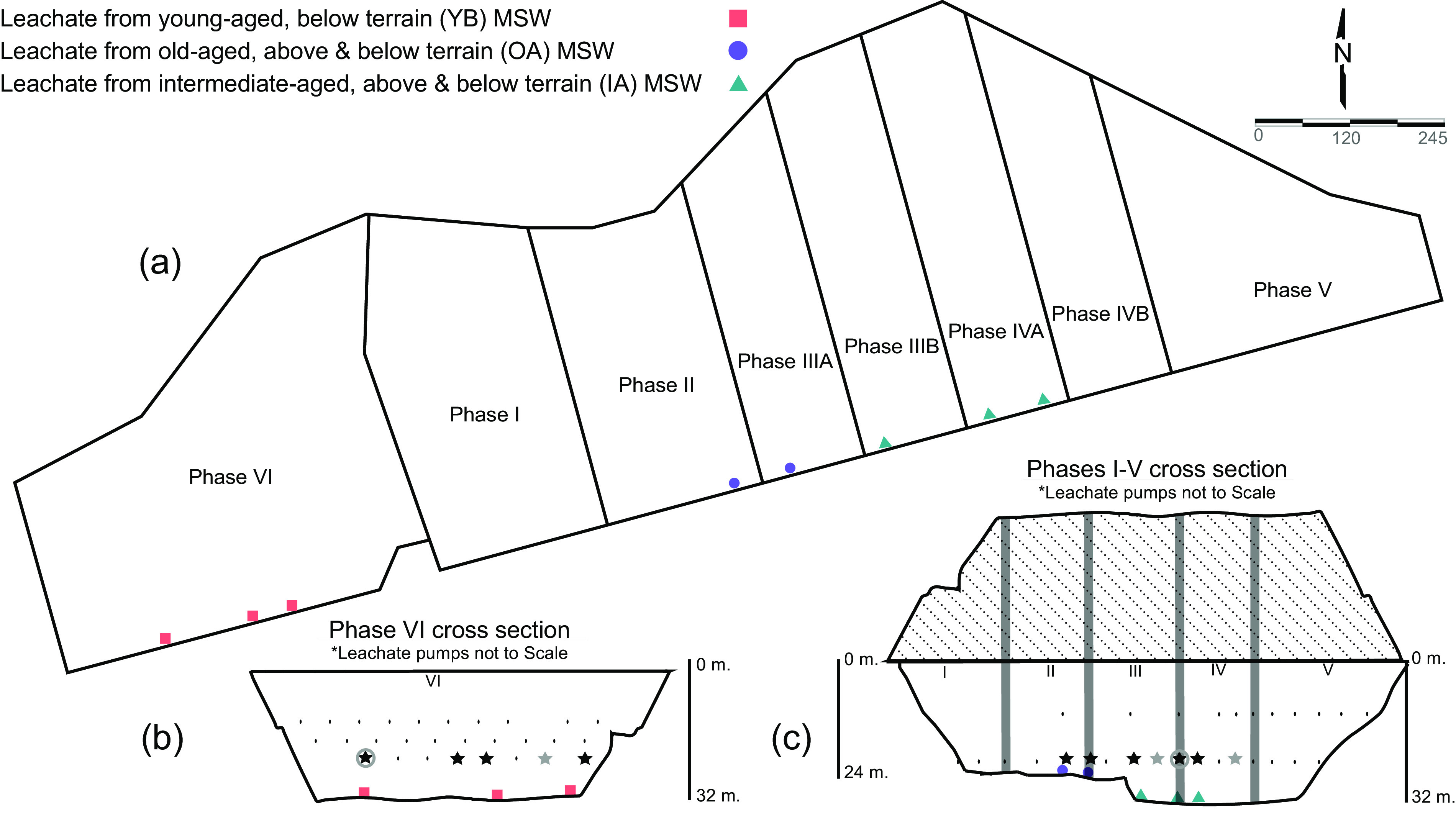
Maps of the arid Salt River Landfill (SRL), including leachate and gas sampling locations. Aerial schematic view presenting the locations of leachate pumps (*n* = 8) sampled in the drainage network in 2018 from phase VI (west) to phase V (east) (a). Cross sections of defining sections, phase VI (b) and phases I to V (c), depicting cell depths (32 m); a subset of below-terrain, horizontal gas extraction wells (*n* = 56) and their placement (dots); and above-terrain MSW disposal (gray fill) (b and c). Select gas samples (denoted with stars) were used in carbon stable-isotope analysis. Gray stars indicate gas that was collected in 2018 (*n* = 6), and black stars represent samples from May 2020 (*n* = 8). Gas samples collected on both samplings are depicted as black, circumscribed stars.

We studied the arid Salt River Landfill (SRL) located in Scottsdale, AZ. SRL is a 143-acre site delineated along six phases containing eight cells dug below the terrain surface (24 to 32 m) accumulating MSW up to or above terrain level. SRL was constructed in two distinct sections (Fig. 8): the oldest-buried MSW, which encompasses phases I to V (107.7 acres), beginning in 1993, and the more recently buried MSW forming the remainder of the site, phase VI (35.3 acres), in 2009. Phases I to V also experienced active, above-terrain surface MSW disposal, which has not yet been implemented in phase VI. As of 2018, SRL has received approximately 1,600 tons of MSW daily.

Gas and leachate samples were categorized by the time since the initial MSW disposal within the landfill cell and whether above-terrain MSW disposal was active during our sampling. Thus, gas and leachate by-products collected in this study were assigned cell categories that were used when referring to leachate and/or gas samples: young-aged, below-terrain MSW (YB); intermediate-aged, above- and below-terrain MSW (IA); and old-aged, above- and below-terrain MSW (OA). YB gas and leachate were collected from phase VI, IA gas and leachate were collected from phases IIIB and IVA, and OA gas and leachate were collected from phases II and IIIA. Phases I, IVB, and V did not yield leachate during sampling and, hence, were not included in our 2018 analysis.

### Leachate sample collection and processing.

Five-hundred-milliliter leachate samples (*n* = 8) were collected in duplicate directly from pumps on 12 June 2018 from 5 cells (Fig. 8). Samples were stored on ice immediately after collection and processed for biomass collection within 72 h using an autoclave-sterilized vacuum filtration device. A summary of the 2018 leachate sampling size and corresponding leachate samples is included in Table S5 in the supplemental material.

### Leachate chemical analyses.

Leachate pH, conductivity, and temperature were measured on-site using a portable probe (Oakton, USA). Organic and inorganic geochemical characterization of leachate was performed according to various standard techniques (Table S1). Filtered leachate was also screened for organic acid production by high-performance liquid chromatography (HPLC). The HPLC instrument (Shimadzu, Japan) used an Aminex HPX-87H column (Bio-Rad, USA) with 5 mM sulfuric acid as the mobile phase under a flow rate of 0.6 mL min^−1^ and an oven temperature of 50°C. Signals were collected from a UV-visible (UV-Vis) detector at 210 nm. The identities and concentrations of analytes were determined from multipoint analytical calibration standards.

### DNA extraction, PCR amplification, and next-generation sequencing (NGS).

The microbial biomass in leachate was concentrated on a mixed-cellulose-ester filter (0.2-μm pore size; Whatman, UK). One-quarter of this filter was used for genomic DNA extraction with a NucleoSpin soil kit (Macherey-Nagel, Germany). PCR amplification was performed in triplicate 25-μL reaction mixtures using unique, barcoded pairs of the 515F (76) and 909R (77) primers targeting the V3-V4 region of the 16S rRNA gene of both *Bacteria* and *Archaea*. For amplification of the *mcrA* functional gene, the *mcrA*-mlas and *mcrA*-rev primers were used (78). PCR products were normalized, purified, and pooled prior to paired-end sequencing in a multiplexed amplicon library under the Illumina MiSeq 2-by-300 V3 module.

### Bioinformatic processing of NGS reads.

Forward and reverse reads were merged through the context-aware scheme for paired-end reads from high-throughput amplicon sequencing (CASPER) tool (79). An in-house python script demultiplexed the amplicon library (https://github.com/Hinsby/Manta_Illumina_amplicon_demultiplexing). Next, 16S rRNA amplicons were trimmed to a common length (355 nucleotides), followed by assembly into absolute sequence variants (ASVs) through the Deblur standalone tool using default parameters (80). ASVs were assigned taxonomy using the SILVA 132 database via a naive Bayes taxonomic classifier trained on the 16S primers and amplicon lengths (355 nucleotides) used in this study through Qiime2 (81) version 2017.12. For analysis of the *mcrA* amplicons, the Ribosomal Database Project Fungene pipeline (82) was used with a customized amino acid *mcrA* database. The *mcrA* database used (224 sequences) is provided in Data Set S2. Merged *mcrA* reads were trimmed to a common length (420 nucleotides) before being translated into amino acids, sequence error corrected, and assigned taxonomy through nearest-neighbor classification using the FrameBot tool (83). Dereplicated and chimera-checked *mcrA* reads were clustered at 86% similarity, representing a species phylogenetic rank of this functional marker gene (84). Qiime2 and the R package phyloseq (85) were used to investigate alpha and beta diversity indices on a rarefied abundance table for the 16S rRNA (6,380 sequences) and *mcrA* (2,970 sequences) data sets, respectively. Differential abundance testing was performed on unrarefied abundance tables at diverse phylogenetic ranks by the analysis of the composition of microbiomes (ANCOM) Qiime2 plug-in (86). When appropriate, follow-up testing via Kruskal-Wallis testing was performed for groups presented in Table S2. For comparison of significant beta diversity by cell category, PERMANOVA/Adonis PERMANOVA was run on unweighted UniFrac (16S rRNA gene) and Bray-Curtis (*mcrA* gene) distance matrices. Negative controls (*n* = 4) represented by water blanks associated with sample collection and the filters used for biomass collection yielded negligible average counts of 16S and *mcrA* sequencing reads per sample postprocessing. Accordingly, negative-control samples and their reads were removed from the data sets.

Ecological niche breadth estimates (Tables S3 and S4) were calculated on *mcrA* sequence data using Feinsinger’s proportional similarity derived from the R package MicroNiche on geochemical parameters acting as the primary source of dissimilarity of the methanogenic community in ordination clusters of our CCAs (i.e., leachate temperature and TDS) (Fig. 5). Although propionate is the strongest contributor to CCA1, select samples had nondetectable values in this data set, preventing it from being an appropriate input.

### Landfill gas concentration analysis.

A gas emission monitor (Landtec, USA) was connected to an outlet port of the gas extraction well header to analyze gas composition (e.g., CH_4_, CO_2_, O_2_, CO, and H_2_S), temperature, and flow rate. Gas samples (stars in Fig. 8) were directly injected into either 30-mL glass serum vials equipped with a butyl stopper and a metal crimp top on 11 June 2018 or Tedlar gas bags on 7 and 29 May 2020. Gas collected in Tedlar bags was quickly transferred to glass vials within 3 to 4 h. Gray stars in Fig. 8 indicate gas that was collected in 2018 (*n* = 6), and black stars represent samples from 2020 (*n* = 8). Gas samples collected on both samplings are depicted as black, circumscribed stars.

Monthly gas concentration readings were analyzed for each leachate sampling event (October 2016 to 2018) and the month of May 2020, excluding gas from leachate cleanout (phase VI only), above terrain (phases I to V only), and outlier gas extraction wells (possessing <35% CH_4_). A summary of the 2018 gas sampling size is included in Table S5.

### Stable isotopic estimation of hydrogenotrophic methanogenic metabolism.

Measurements of carbon stable isotopic ratios for CH_4_ (δ^13^C of CH_4_) and CO_2_ (δ^13^C of CO_2_) from gas samples were conducted to estimate hydrogenotrophic methanogenic activity. Stable-isotope ratios were measured using a cavity ring-down spectrometry instrument (G2201-i) in the dual-high-range mode (Picarro, USA). The delta values, reported in reference to the standard Pee Dee belemnite, were input as variables in calculations of fractionation factors.

### Statistical analysis.

All statistical tests were performed using Prism software version 9.0.0 (GraphPad, USA). Nonparametric Kruskal-Wallis tests were conducted due to the unequal sample sizes in most of our analyses but on Gaussian-distributed data sets (*P* > 0.05 by a Shapiro-Wilk test). Unpaired, two-way Student’s *t* tests or Mann-Whitney U tests were used to compare only two cell categories when an analyte was undetectable below instrument detection limits (e.g., propionate) or not measured due to low leachate yields during sampling (e.g., OA leachate). Pairwise comparisons were conducted using Dunnett’s multiple-comparison test when statistical outcomes were observed. The standard error of the mean was used as the variance calculation in all descriptive statistics.

### Data availability.

16S rRNA and *mcrA* gene sequences were deposited in the NCBI Sequence Read Archive under BioProject accession numbers PRJNA741046 and PRJNA748531, respectively (BioSample accession numbers SAMN19843592 to SAMN19843611). Summarized measurements (geochemistry, HPLC, and carbon stable-isotope data) are publicly available (https://figshare.com/s/6adb2895c59fae62caf3). The python script and guiding bash script for demultiplexing Illumina paired-end sequences are available at https://github.com/Hinsby/Manta_Illumina_amplicon_demultiplexing.
