# Supplementary material for: Delineating the Drivers and Functionality of Methanogenic Niches within an Arid Landfill
Source: Appl Environ Microbiol. 2022 Apr 11;88(9):e02438-21. doi: 10.1128/aem.02438-21 (PMC9088289; doi:10.1128/aem.02438-21)
Supplement: Supplemental file 1 — Fig. S1 to S8, Tables S1 to S5, and descriptions of other supplemental content. Download aem.02438-21-s0001.pdf, PDF file, 0.6 MB [file aem.02438-21-s0001.pdf]

MS Title:" Delineating the drivers and functionality of methanogenic niches within an arid landfill"

Authors: Mark C. Reynolds [a,b,c], Damien Finn* [a,b], Analissa F. Sarno [a,b], Richard Allen [c,d], J. David Deathrage [e], Rosa Krajmalnik-Brown [b,c,f], Hinsby Cadillo-Quiroz [a,b,c] #

#Correspondence:
hinsby@asu.edu

AFFILIATIONS
[a] School of Life Sciences, Arizona State University, Tempe, AZ, United States of America
[b] Biodesign Institute, Arizona State University, Tempe, AZ, United States of America
[c] Center for Bio-mediated & Bio-inspired Geotechnics, Arizona State University, Tempe, AZ, United States of America
[d] Salt River Landfill, Scottsdale, AZ, United States of America
[e] Copper State Engineering Inc., Scottsdale, AZ, United States of America
[f] School of Sustainable Engineering for the Built Environment, Arizona State University, Tempe, AZ, United States of America

SUPPLEMENTARY MATERIAL

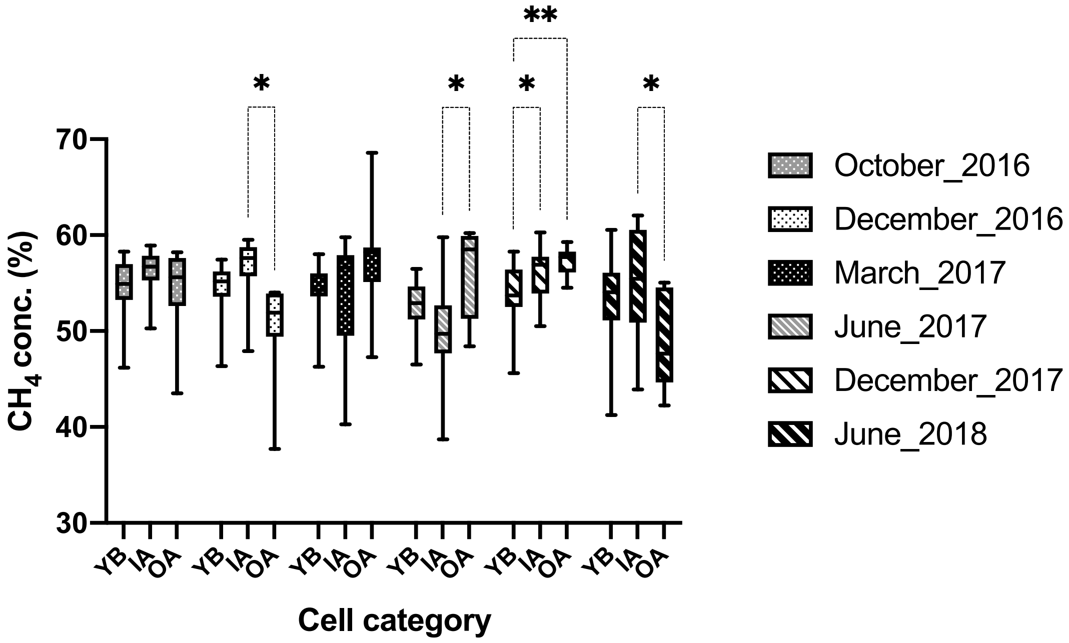

FIG S1. CH_4 concentration (n=336; nonparametric mixed effects model) across distinct landfill cell categories. Monthly values for all samplings between 2016 and 2018 are shown. **Key** – Cell categories: young-aged, below terrain MSW (**YB**), intermediate-aged, above & below terrain MSW (**IA**), and old-aged, above & below terrain MSW (**OA**). Calculated p-value: ≤0.05(*****), ≤0.002(**).

---

* Present address: Thünen Institut für Biodiversität, Johann Heinrich von Thünen Institut, Braunschweig, Germany 38116

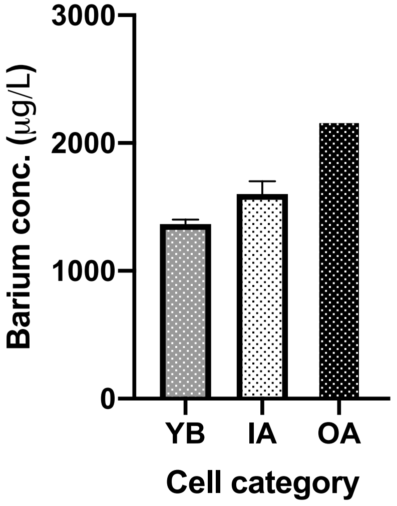

FIG S2. June 2018 leachate barium concentrations (n=7, Kruskal Wallis) across distinct landfill cell categories. **Key** – Cell categories: young-aged, below terrain MSW (**YB**), intermediate-aged, above & below terrain MSW (**IA**), and old-aged, above & below terrain MSW (**OA**).

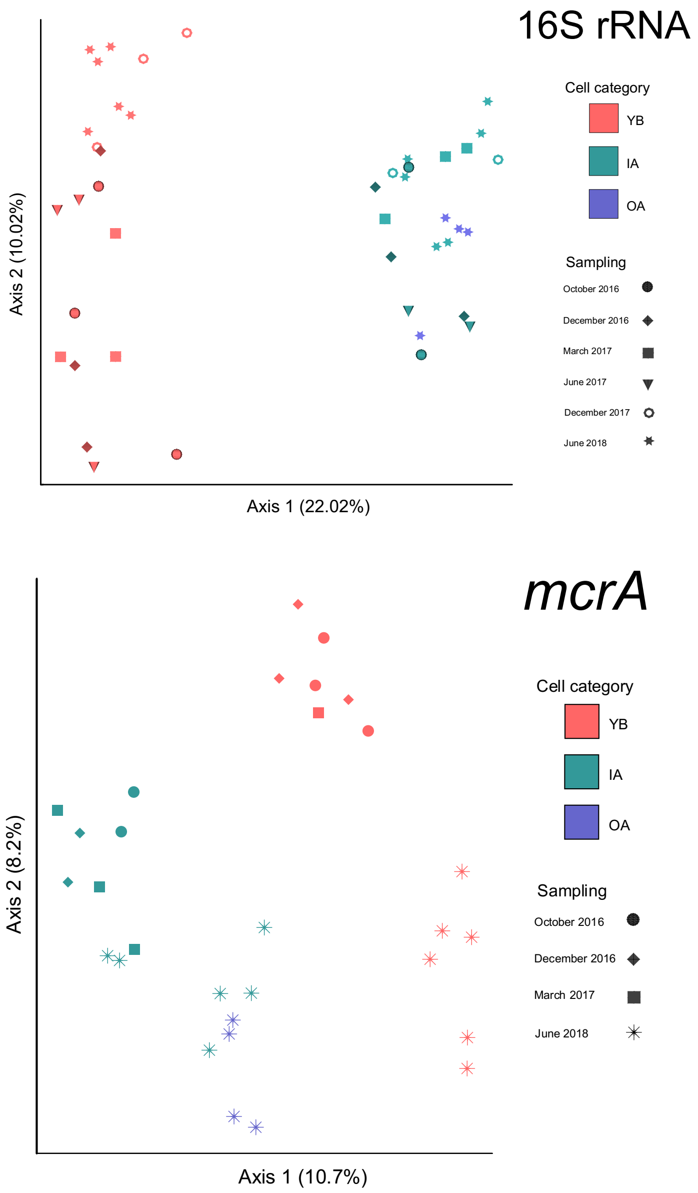

FIG S3. Principal coordinate analysis (PCoA) ordination using unweighted UniFrac distances from (top) 16S rRNA gene and (bottom) *mcrA* abundance data combining data from all samplings between 2016-2018. **Key** – Cell categories: young-aged, below terrain MSW (**YB**), intermediate-aged, above & below terrain MSW (**IA**), and old-aged, above & below terrain MSW (**OA**).

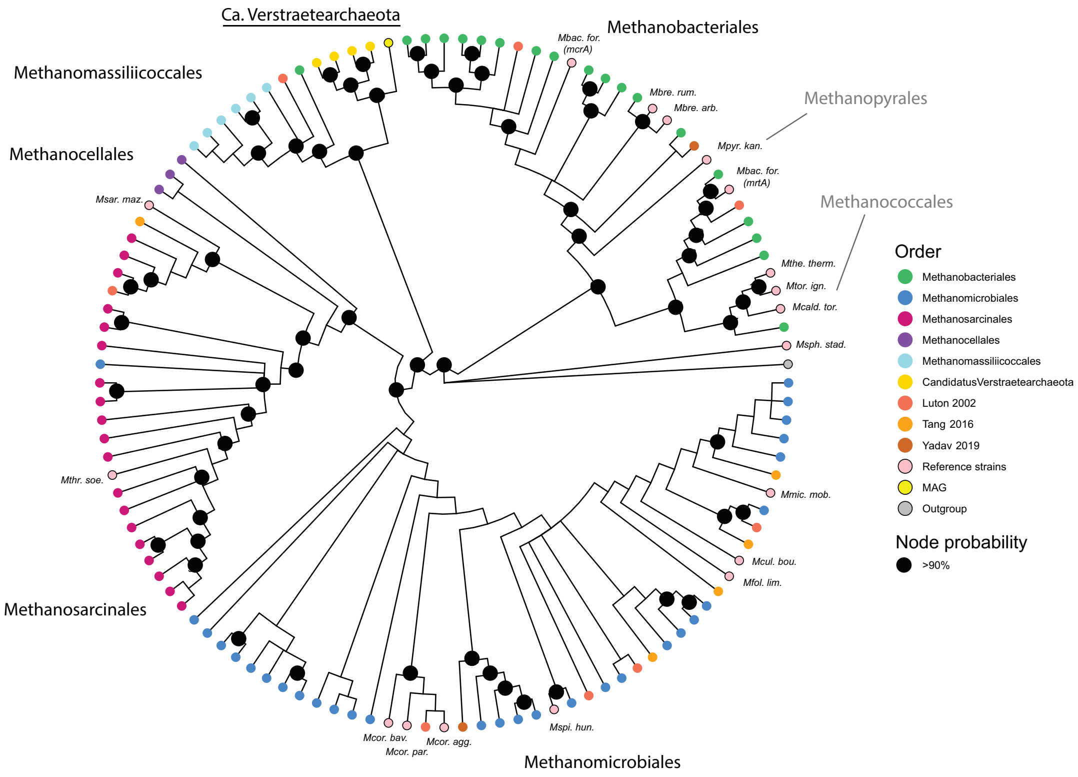

FIG S4. Bayesian inference phylogeny of representative *mcrA* sequences recovered from SRL leachate during samplings between 2016-2018. Also included are environmental sequences clustered at 72% similarity from three methanogenic leachate community studies (n=15) (1–3), select sequences from references strains (n=18, (1), and a single, putative *mcrA* sequence Candidatus *Verstraetearchaeota* metagenome assembled genome (MAG) (4). Briefly, representative sequences (n=1,673) were first clustered at 72% similarity, followed by retention of 81 sequences representing highly abundant clusters with no substantial gaps (<3 gaps) (note: these abundant sequences represent 92% of total reads in the combined dataset). Black circles denote greater than 90% model posterior probability for node agreement. **Key** – Reference strain: *Msph. stad.* (*Methanosphaera stadtmanae* DSM 3091), *Mmic. mob.* (*Methanomicrobium mobile* BP), *Mcue. bou.* (*Methanoculleus bourgensis MS2), Mfol. lim* (*Methanofollis liminatans DSM 4140*), *Mspi. hun.* (*Methanospirillum hungatei* JF-1), *Mcor. agg.* (*Methanocorpusculum aggregans*), *Mcor. par.* (*Methanocorpusculum parvum), Mcor. bav.* (*Methanocorpusculum bavaricum*), *Mthr. soe.* (*Methanothrix soehngenii* GP6), *Msar. maz.* (*Methanosarcina mazei*), *Mbac. for. (mcrA)* (*Methanobacterium formicicum* methyl coenzyme reductase I subunit A), *Mbre. rum.* (*Methanobrevibacter ruminantium* M1), *Mbre. arb.* (*Methanobrevibacter arboriphilus* JCM 13429), *Mpyr. kan.* (*Methanopyrus kandleri*), *Mbac. for. (mrtA)* (*Methanobacterium formicicum* methyl coenzyme reductase II subunit A), *Mthe. therm.* (*Methanothermococcus thermolithotrophicus* DSM 2095), *Mtor. ign.* (*Methanotorris igneus* Kol 5), & *Mtor. ign.* (*Methanocaldococcus jannaschii* DSM 2661). Methanogenic order: Black text *(recovered in this dataset)*. Grey text *(not recovered in this dataset)*. Methanogenic phylum: Non-underlined text (*Euryarchaea).* Underlined text (*Non-Euryarchaea*). Outgroup: *Methanopyrus kandleri* AV19 Tetrahydromethanopterin S-methyltransferase subunit A.

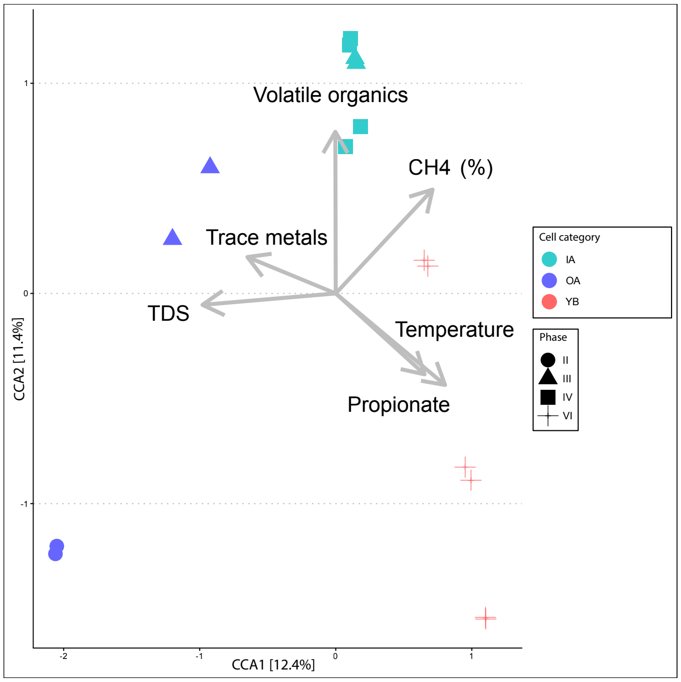

FIG S5. Canonical correspondence analysis (CCA) ordination of 16S rRNA gene abundance data from June 2018 to visualize landfill niches using Bray-Curtis distances.

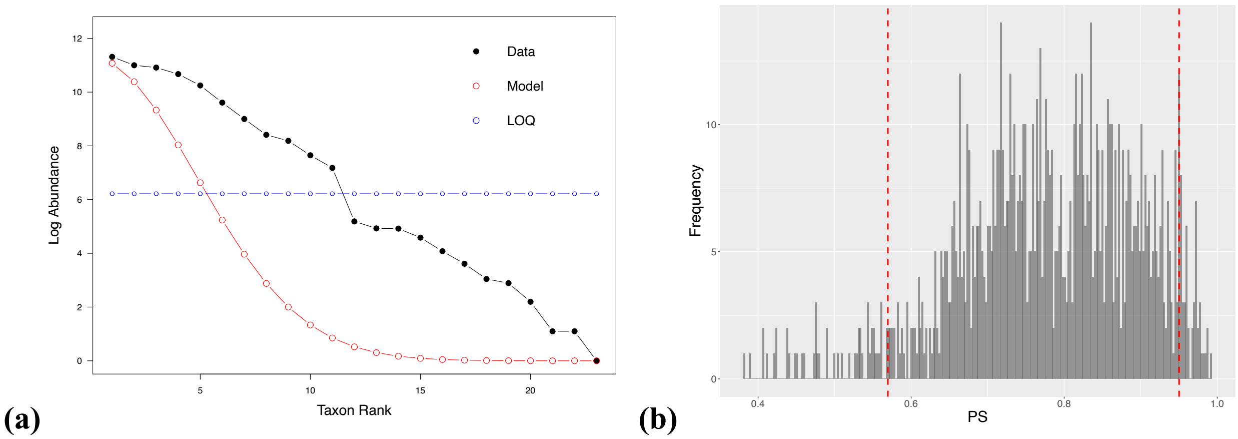

FIG S6. MicroNiche proportional similarity TDS analysis from the *mcrA* gene abundance table. (a) null hypothesis model and distribution and (b) niche breadth calculations. Families (n=12) were flagged as potential false positives if their $\log_{10}$ abundances were below the limit of quantification (LOQ).

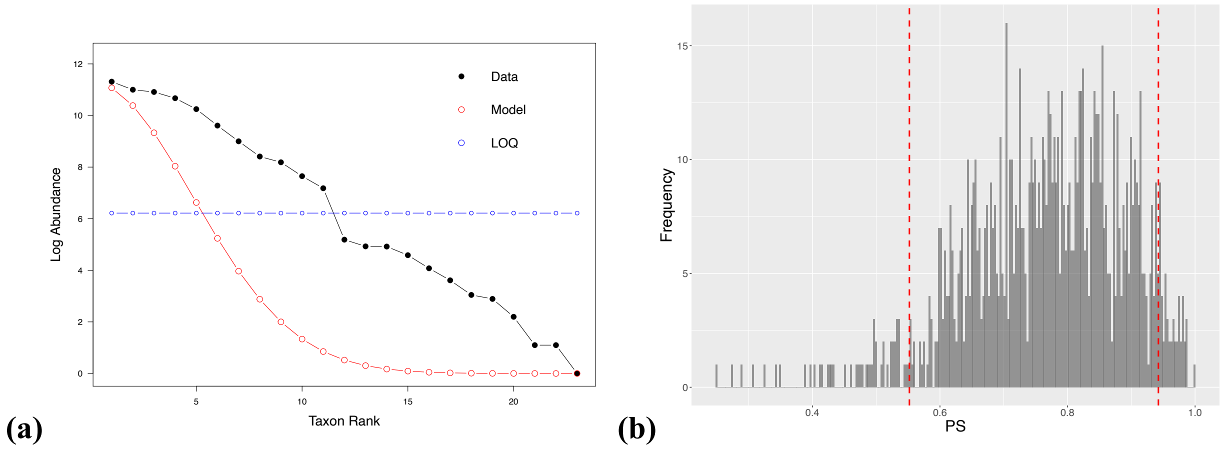

FIG S7. MicroNiche proportional similarity temperature analysis from the *mcrA* gene abundance table. (a) null hypothesis model and distribution and (b) niche breadth calculations. Families (n=12) were flagged as potential false positives if their $\log_{10}$ abundances were below the limit of quantification (LOQ).

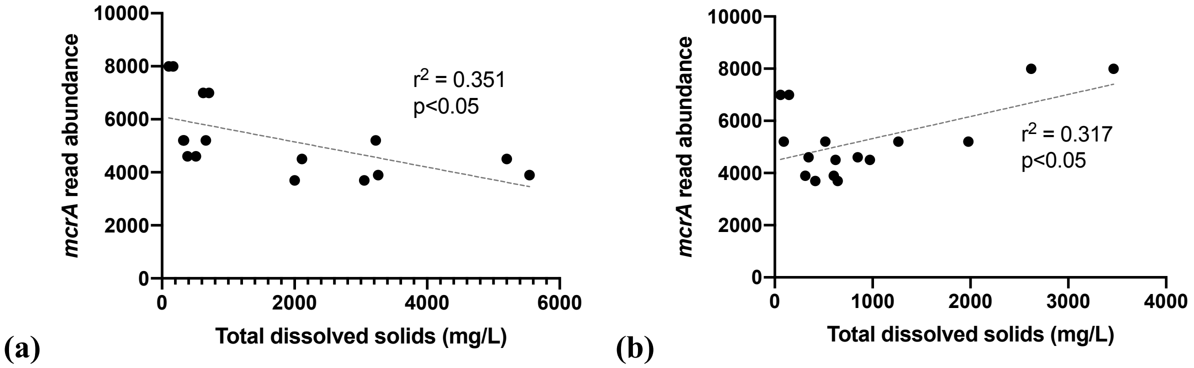

FIG S8. Simple linear regression of (a) *Methanocalculacea* & (b) Candidatus *Verstraetearchaeota mcrA* read abundances against total dissolved solid concentrations yields a significant, negative and positive correlation, respectively.

Analyte/Measurement	Standard Method	Sample analyte min-max (2018)
Volatile organics	EPA 8260B	71.65-310.3 μg/L
Total dissolved solids (TDS)	SM2540 C	3700-8000 mg/L
Total alkalinity*	SM2320 B	600.0-860.0 mg/L
Ammonia as N*	EPA 350.1	9.300-32.00 mg/L
Biological oxygen demand (BOD)*	SM5210	14.00-29.00 mg/L
Chemical oxygen demand (COD)*	EPA 410.4	890.0-1300 mg/L
Trace metals	EPA 200.8	1651-3946 μg/L
Dissolved organic carbon*	SM 5310B	86.00-140.0 mg/L
pH	N.A.+	6.44-6.76
Conductivity	N.A.+	2830-4410 μS/cm
Temperature	N.A.+	35.50-45.10°C

*OA samples were not included in these analyses in 2018's sampling.
+Probe-based measurements used instead of Standard Methods.

TABLE S1. Environmental Protection Agency (EPA) and other Standard Methods (SM) used for the geochemical characterization of leachate.

**Taxonomic assignment**	**YB%** (±)	**IA%** (±)	**OA%** (±)	**ANCOM test statistic** (W)	**Statistical pairwise comparison** (test used)
**Rhodocyclaceae**	**10.38** (1.84)	**0.292** (0.105)	**0** (0)	328	**YB** vs. IA (Student's t-test)
**Uncultured Omnitrophica bacterium** (OP3)	**7.71E-03** (4.10E-03)	**15.6** (6.3)	**5.05** (1.53)	346	YB vs. **IA** (Kruskal Wallis)
**Marine Benthic Group D and DHVEG-1** (Izemarchaea)	**0** (0)	**0.298** (0.105)	**15.76** (8.20)	326	**OA** vs. IA (Mann Whitney test)
**Unassigned**	**0** (0)	**0.729** (0.223)	**14.66** (5.04)	344	**OA** vs. IA (Mann Whitney test)

TABLE S2. Relative frequencies of family-level reads identified to be differentially abundant according to cell category using ANCOM. Bolded and underlined font in the "Statistical pairwise comparison" column indicates which cell category contained the highest abundance. **Key** – Cell categories: young-aged, below terrain MSW (**YB**), intermediate-aged, above & below terrain MSW (**IA**), and old-aged, above & below terrain MSW (**OA**).

Taxa	Feinsinger's PS	p-value	Adj. p-value	Below LOQ?
**Methanosarcinaceae**	**0.86334834**	**0.37135147**	**0.53381774**	**N**
**Methanomicrobiaceae**	**0.85442**	**0.42378105**	**0.57335083**	**N**
**Methanotrichaceae**	**0.78761626**	**0.92347474**	**0.94013372**	**N**
**Candidatus Verstraetearchaeota**	**0.78562721**	**0.94013372**	**0.94013372**	**N**
**Methanomassiliicoccaceae**	**0.7600507**	**0.84589128**	**0.92645235**	**N**
Methanocaldococcaceae	0.74825014	0.74996156	0.8624558	Y
**Methanocorpusculaceae**	**0.73599498**	**0.65429308**	**0.79203899**	**N**
**Methanoregulaceae**	**0.71022223**	**0.47193211**	**0.60302436**	**N**
Methanobacteriaceae_WP_069583233.1	0.69260827	0.36551119	0.53381774	Y
Unclassified Methanomassiliicoccales_WP_177971605.1	0.67937054	0.29630884	0.4867931	Y
**Methanobacteriaceae**	**0.65514828**	**0.19374528**	**0.34278011**	**N**
Methanobacteriaceae_WP_048081846.1	0.6520727	0.182866	0.34278011	Y
**Methanospirillaceae**	**0.65075524**	**0.17834683**	**0.34278011**	**N**
Unclassified Methanomassiliicoccales_WP_178196165.1	0.63855422	0.14037168	0.32285486	Y
**Methanocalculaceae**	**0.54104335**	**0.01235815**	**0.03158193**	**N**
**Methanocellaceae**	**0.50540379**	**0.00401177**	**0.01153383**	**N**
Candidatus Nezhaarchaeota	0.43443848	0.00028907	0.00094981	Y
Candidatus Methanoperedenaceae	0.36144578	1.11E-05	4.27E-05	Y
Methanobacteriaceae_WP_048080940.1	0.34698795	5.46E-06	2.51E-05	Y
Methanosarcinaceae_WP_048120720.1	0.34698795	5.46E-06	2.51E-05	Y
Archaeoglobi	0.3133054	9.53E-07	7.31E-06	Y
Candidatus Methanodesulfokores	0.29156627	2.90E-07	3.33E-06	Y
Candidatus Methanomethylophilaceae	0.29156627	2.90E-07	3.33E-06	Y

TABLE S3. MicroNiche proportional similarity TDS results from the *mcrA* gene abundance table. When a taxa's p-value <0.05, its niche breadth is significantly different than the mean of the null model. An adjusted p-value (Benjamin-Hochberg) accounts for false discovery rates inherent in microbial amplicon sequencing data. Families in bold text (n=11) had $\log_{10}$ abundances above the limit of quantification (LOQ) and were plotted in Fig. 7a and 7b (in manuscript), while maroon text represent families with significantly variable, calculated niche breadths.

Taxa	Feinsinger's PS	p-value	Adj. p-value	Below LOQ?
**Methanomicrobiaceae**	**0.95650724**	**0.04609534**	**0.1177992**	**N**
**Methanosarcinaceae**	**0.94252538**	**0.06471652**	**0.148848**	**N**
**Methanomassiliicoccaceae**	**0.88161484**	**0.22801848**	**0.38119774**	**N**
**Methanotrichaceae**	**0.84641528**	**0.40392691**	**0.51612883**	**N**
Methanobacteriaceae_WP_069583233.1	0.82447704	0.54617953	0.66116469	Y
**Methanoregulaceae**	**0.82033501**	**0.57557776**	**0.66191443**	**N**
unclassifiedMethanomassiliicoccales_WP_177971605.1	0.81123932	0.64262858	0.6984211	Y
unclassifiedMethanomassiliicoccales_WP_178196165.1	0.77042299	0.97287709	0.97287709	Y
Methanocaldococcaceae	0.72649415	0.66805496	0.6984211	Y
**Methanobacteriaceae**	**0.67690427**	**0.34146187**	**0.46197783**	**N**
Methanobacteriaceae_WP_048081846.1	0.67382869	0.3252709	0.46197783	Y
**Methanospirillaceae**	**0.67251123**	**0.31849068**	**0.46197783**	**N**
**Candidatus Verstraetearchaeota**	**0.65375844**	**0.23203341**	**0.38119774**	**N**
**Methanocalculaceae**	**0.65115612**	**0.22149807**	**0.38119774**	**N**
**Methanocorpusculaceae**	**0.6258822**	**0.13653171**	**0.2854754**	**N**
**Methanocellaceae**	**0.52715979**	**0.01144086**	**0.03289248**	**N**
Archaeoglobi	0.42341817	0.00029242	0.00096081	Y
Candidatus Methanomethylophilaceae	0.40167904	0.00011766	0.00045102	Y
Candidatus Methanodesulfokores	0.40167904	0.00011766	0.00045102	Y
Methanobacteriaceae_WP_048080940.1	0.36874395	2.69E-05	0.00015487	Y
Methanosarcinaceae_WP_048120720.1	0.36874395	2.69E-05	0.00015487	Y
Candidatus Nezhaarchaeota	0.30256971	9.82E-07	1.13E-05	Y
Candidatus Methanoperedenaceae	0.22957701	1.48E-08	3.40E-07	Y

TABLE S4. MicroNiche proportional similarity temperature results from the *mcrA* gene abundance table. When a taxa's p-value <0.05, its niche breadth is significantly different than the mean of the null model. An adjusted p-value (Benjamin-Hochberg) accounts for false discovery rates inherent in microbial amplicon sequencing data. Families in bold text (n=11) had $\log_{10}$ abundances above the limit of quantification (LOQ) and were plotted in Fig. 7a and 7b (in manuscript), while maroon text represent families with significantly variable, calculated niche breadths.

Leachate sampling date	Leachate sample	Cell category	2018 Analytes measured	Gas & Isotope sampling date	2018 Gas sample size[#]	2018 Isotope sample size	2020 Isotope sampling date	2020 Isotope sample size
6/12/2018	LPA	YB	ALL	6/11/2018	6	1	5/29/2020	1
6/12/2018	LPB	YB	ALL	6/11/2018	11	1	5/29/2020	1
6/12/2018	LPC	YB	ALL	6/11/2018	11	1	5/07/2020	1
6/12/2018	LP8	IA	ALL	6/11/2018	21	1	5/29/2020	1
6/12/2018	LP10	IA	ALL[+]	6/11/2018		1	5/07/2020	1
6/12/2018	LP11	IA	ALL[+]	6/11/2018		0[^]	5/29/2020	1
6/12/2018	LP5	OA	-COD<br>-BOD<br>-NH_3.<br>-Alk.<br>-DOC	6/11/2018	7	0	5/07/2020	1
6/12/2018	LP6	OA	-COD<br>-BOD<br>-NH_3.<br>-Alk.<br>-DOC	6/11/2018		0	5/07/2020	1

[-]Negative sign preceding an analyte's name indicates it was not measured.
[+]Studied as composite samples for all analytes except pH, conductivity, and temperature due to low leachate yield.
[^]One of six gas samples (13L) is more proximal to leachate samples LP12 and/or LP13, which did not yield leachate throughout our study period.
[#]Phase VI (YB samples) was constructed as a bioreactor landfill cell. Hence, the specific affiliation of gas samples to leachate samples due to ~2x more gas wells present in Phase VI.

TABLE S5. Leachate and gas sampling details, and sample sizes, including or lacking if select analyte measurements were obtained. **Key** – COD (chemical oxygen demand), BOD (biological oxygen demand), NH_3 (ammonia as N), Alk. (alkalinity), DOC (dissolved organic carbon).

Supplementary files legends:

Supplemental File 2 (not shown here) is a .fasta file containing the custom, amino acid *mcrA* database used for taxonomic classification through Framebot in the FunGene Pipeline.

Detailed mcrA database preparation. The custom *mcrA* database was prepared from National Center for Biotechnology's RefSeq Protein database (5) using the R package reutils, with search query of "coenzyme-B sulfoethylthiotransferase subunit alpha", yielding total of 770 semi-redundant, taxonomic lineages with both Euryarchaeal, non-Euryarchaeal, and unclassified archaeal sequences. Sequences were aligned and initial curation was conducted to remove identical, incomplete, or short sequences, followed by retention of sequences that were curated through the updated NCBI's prokaryotic genome annotation pipeline (PGAP) (6). Select sequences that were not annotated through PGAP were included if they had >70 bootstrap node values, tight placement within their corresponding genera, or taxa we previously observed classified in the preliminary study of SRL. Maximum likelihood phylogeny was conducted in RAxML (version 8.2.12) to assess curation efforts.

Supplemental File 3 (not shown here) is a .xlsx file with FrameBot hits (classified as species rank, but with assignment truncated to family rank) against the custom, amino acid *mcrA* database and their confidence scores.

References:

1. Luton PE, Wayne JM, Sharp RJ, Riley PW. 2002. The mcrA gene as an alternative to 16S rRNA in the phylogenetic analysis of methanogen populations in landfill. Microbiology 148:3521–3530.
2. Tang W, Wang Y, Lei Y, Song L. 2016. Methanogen communities in a municipal landfill complex in China. FEMS Microbiol Lett 363:1–7.
3. Yadav S, Maitra S, Ghosh S. 2019. Cloning and Sequencing of Methyl-coenzyme Reductase A (mcrA) Gene of Methanogenic Archaea from Landfill. Sci Technol J.
4. Wang Y, Wegener G, Hou J, Wang F, Xiao X. 2019. Expanding anaerobic alkane metabolism in the domain of Archaea. Nat Microbiol. Springer US.
5. Haft DH, DiCuccio M, Badretdin A, Brover V, Chetvernin V, O'Neill K, Li W, Chitsaz F, Derbyshire MK, Gonzales NR, Gwadz M, Lu F, Marchler GH, Song JS, Thanki N, Yamashita RA, Zheng C, Thibaud-Nissen F, Geer LY, Marchler-Bauer A, Pruitt KD. 2018. RefSeq: An update on prokaryotic genome annotation and curation. Nucleic Acids Res 46:D851–D860.
6. Tatusova T, Dicuccio M, Badretdin A, Chetvernin V, Nawrocki EP, Zaslavsky L, Lomsadze A, Pruitt KD, Borodovsky M, Ostell J. 2016. NCBI prokaryotic genome annotation pipeline. Nucleic Acids Res 44:6614–6624.
